# Vaccination Status and Influencing Factors of Delayed Vaccination in Toddlers Born to Hepatitis B Surface Antigen-Positive Mothers

**DOI:** 10.3390/vaccines13030286

**Published:** 2025-03-07

**Authors:** Jinling Gao, Lin Luan, Yiheng Zhu, Jie Zhu, Zhiyuan Zhu, Tian Gong, Juan Xu, Na Liu

**Affiliations:** 1 Suzhou Center for Disease Control and Prevention, Suzhou 215000, China; gaojinling_ss@163.com (J.G.);; 2 Suzhou Municipal Health Commission, Suzhou 215000, China; 3 Suzhou Health and Family Planning Statistics Information Center, Suzhou 215000, China; 4 Suzhou Maternal and Child Health Care and Family Planning Service Center, Suzhou 215000, China; 5 Chinese Center for Disease Control and Prevention, Beijing 100050, China

**Keywords:** HBsAg-positive mother, hepatitis B vaccine, Bacillus Calmette–Guérin, delayed vaccination, influencing factor

## Abstract

**Background**: This study aims to analyze the vaccination status and factors influencing delayed vaccination among toddlers born to hepatitis B surface antigen (HBsAg)-positive mothers. **Methods**: Data of HBsAg-positive mothers between 1 January 2021 and 31 December 2022 were provided by the Suzhou Maternal and Child Health Care and Family Planning Service Center. The vaccination records were obtained from the Jiangsu Province Immunization Service Management Information System. Logistic regression analysis was used to analyze influencing factors of delayed vaccination. **Results**: A total of 4250 toddlers born to HBsAg-positive mothers were documented. The data revealed that the first dose of the hepatitis B vaccine was administered to 100% of the toddlers. In addition, the coverage of the National Immunization Program (NIP) vaccines among these toddlers ranged from 92.9% to 99.4%. The proportion of delayed NIP vaccination varied between 0% and 12.2%. The proportion of delayed Bacillus Calmette–Guérin (BCG) vaccination was 11.3%, with the delay predominantly observed between 4 and 6 months. Notably, the proportion of delayed BCG vaccination among the toddlers born to HBsAg-positive mothers was significantly higher than that in the general population. Additionally, the proportion of the first dose of non-NIP vaccines was 3.3–36.4%, and the proportion of DTaP-IPV/Hib was 27.0%. Logistic regression analysis revealed that the regional level, the mother’s human papillomavirus (HPV) vaccination status, and the infant’s birth weight were significant factors influencing the timeliness of vaccination. **Conclusions**: Although the vaccination status of toddlers born to HBsAg-positive mothers in Suzhou city remains stable, the issue of delayed vaccination requires attention. It is essential to continue strengthening targeted vaccine education to reduce vaccine hesitancy and improve the rate of timely vaccination.

## 1. Introduction

Viral hepatitis is becoming an increasingly fatal global health issue, causing approximately 1.3 million deaths annually. This makes it the second leading cause of death from infectious diseases worldwide [1]. The World Health Organization (WHO) reports a concerning trend: while the incidence of hepatitis B virus (HBV) infection is decreasing globally, the mortality rate is on the rise [2].

HBV infection can occur through a variety of routes, such as vertical and horizontal transmission. In lowly endemic areas, sexual transmission and injecting drug use are mode of transmission of HBV infection [3]. In highly endemic areas, mother-to-child transmission is the most common cause [4]. Mother-to-child transmission accounts for 40–50% of new infections, mainly during the perinatal period. The transmission of hepatitis B surface antigen (HBsAg) from positive mothers to infants and young children typically occurs through blood or other body fluids. Therefore, early intervention to interrupt mother-to-child transmission can effectively control the spread of HBV [5,6].

The hepatitis B vaccine (HepB) was incorporated into the immunization schedule in 1992, mandating all newborns to receive one dose of HepB at birth and subsequent doses at one and six months. Children born to HBsAg-positive mothers should receive HepB and hepatitis B immunoglobulin (HBIG) as early as possible at birth. These vaccination strategies have significantly reduced hepatitis B infection rates. A study in China showed a substantial decline in the rate of chronic hepatitis B infection in the general population, with the infection rate in children under five standing at approximately 0.3% [7]. For HBsAg-positive mothers, the risk of mother-to-child transmission ranges between 70% and 90% [8]. Moreover, the positive rates of HBsAg in children born to HBsAg-positive mothers, vaccinated at 24 h, 24–47 h, and 48–96 h post-delivery, were 5.6%, 7.0%, and 16.7%, respectively [9]. As the interval between birth and vaccination increases, the HBV infection rate from mother-to-child transmission also increases. Therefore, it is crucial that children born to HBsAg-positive mothers receive the HepB vaccine as soon as possible [10].

Vaccination is a powerful tool in preventing infectious diseases, making its timely administration crucial. Children born to HBsAg-positive mothers may be in an unstable physiological state, and attention should be paid to the timely HepB vaccination. At the same time, attention should also be paid to timely vaccination with other vaccines, which is the key factor for vaccine-preventable diseases. Bacille Calmette–Guérin (BCG) is a live vaccine that is mainly used to prevent tuberculosis. BCG remains an important public health measure in China, and it is generally recommended that newborns be vaccinated as early as possible at birth. For toddlers born to HBsAg-positive mothers, BCG is as important as the hepatitis B vaccine in the neonatal period. Similarly, after the infant is stable, other vaccines are administered on time as required by national policy. In summary, understanding the factors influencing timely vaccination can improve the vaccination rates and overall situation for children born to HBsAg-positive mothers.

## 2. Materials and Methods

### 2.1. Data Sources

The data for this study were provided by the Suzhou Maternal and Child Health Care and Family Planning Service Center. They included information on cases of HBsAg-positive mothers, delivery details, and basic information about the children, collected from 1 January 2021 to 31 December 2022. The vaccination information was searched in the “Child vaccination” and “Adult vaccination” modules in the Jiangsu province immunization service management information system.

### 2.2. Vaccines Investigated in This Study

This research focused on National Immunization Program (NIP) vaccines, including the hepatitis B vaccine (Hep B), Bacille Calmette-Guérin, diphtheria and tetanus toxoid with pertussis vaccine (DTP), polio vaccine (PV), measles-containing vaccine (MCV), group A meningococcal polysaccharide vaccine (MPV-A), live Japanese encephalitis vaccine (JEV-L), live attenuated varicella vaccine (VarV, which was added into Suzhou EPI in 2018), and hepatitis A vaccine (Hep A), as well as non-NIP vaccines including the diphtheria and tetanus toxoids and acellular pertussis and Hib conjugate vaccine (DTaP-Hib), diphtheria–tetanus–acellular pertussis inactivated poliomyelitis and Haemophilus influenzae type B (DTaP-IPV/Hib), 13-valent pneumococcal conjugate vaccine (PCV13), enterovirus type 71 vaccine (EV71), and human papilloma virus (HPV) vaccines.

### 2.3. Measurement Formula and Description


(1) $$\mathrm{Proportion of vaccination}=\frac{\mathrm{Number of children vaccinated}}{\mathrm{Number of children planned to be vaccinated}}\times 100\%$$

Here, the number of children planned to be vaccinated is equal to the total planned sample size minus the number of children with contraindications.

The information regarding contraindications to vaccination was recorded by the Jiangsu Province Immunization Service Management Information System on the child vaccination information page under the remarks column.

(2) $$\mathrm{Proportion of delayed vaccination}=\frac{Number\;of\;delayed\;vaccinated}{Number\;of\;children\;planned\;to\;be\;vaccinated}\times 100\%$$

Here, the number of delayed vaccinations is equal to the number of children vaccinated as planned minus the number of children vaccinated on time.

According to the *National Immunization Planning Vaccine Children Immunization Procedures and Instructions* (2021) [11], timely vaccination was determined. HepB_1_ should be inoculated within 24 h after birth. BCG should be completed within 3 months of age. HepB_3_, PV_3_, DTP_3_, MCV_1_, and JEV-L_1_ should be vaccinated within 12 months of age. MPV-A_2_ should be vaccinated within 18 months of age. HepA_1_, DTP_4_, and MCV_2_ should be vaccinated within 24 months of age.

### 2.4. Statistical Methods

All statistical analyses were conducted using IBM SPSS version 21.0. The descriptive analysis was performed for sample characteristics. Absolute frequencies and percentages of qualitative variables were calculated and compared using the chi-square test. Timely vaccination was the dependent variable, and logistic regression was used to analyze the influencing factors, *p* < 0.05 was set as the threshold for statistical significance.

## 3. Results

### 3.1. Study Population and Characteristics

A total of 4250 toddlers born to HBsAg-positive mothers were incorporated into this study from 1 January 2021 and 31 December 2022. Among these, ten toddlers exhibited contraindications for BCG, two for PV, one for DTP, four for MCV, four for JEV-L, two for MPV-A, and four for VarV. A total of 98,492 vaccine doses were analyzed.

In terms of gender distribution, the ratio of males to females stood at 1.15:1. In terms of age classification, 2048 (48.2%) of the toddlers were in the 1-year-old group, while 2202 (51.8%) fell into the 2-year-old group. In terms of geographical distribution, 1940 (45.6%) were living in county-level regions, and 2310 (54.4%) were in city-level regions (Table 1).

### 3.2. Vaccination Status of National Immunization Program Vaccines

The vaccination coverage for NIP vaccines among toddlers born to HBsAg-positive mothers ranged from 92.9% to 100% during 2021–2022. The vaccination coverage varied from 94.8% to 100% in the 1-year-old group and between 92.9% to 100% in the 2-year-old group.

Regarding vaccination delays, the proportion of delayed vaccination fluctuated between 0% and 11.9% during 2021–2022. The proportion of delayed vaccination spanned from 0% to 10.3% in the 1-year-old group and from 0% to 12.2% in the 2-year-old group. Specifically, the delay rate for the BCG vaccine stood at 11.3%, which was relatively high for delayed vaccination, as BCG was required by policy to be given as soon as possible at birth. Additional baseline information is provided in Table 2.

### 3.3. Analysis of Delay in BCG Vaccination

In the general pediatric population, the proportion of delayed BCG vaccinations among children born in 2021–2022 was 5.2%. There were 10,467 instances of BCG vaccination delays, including 4775 in the 1-year-old group and 5692 in the 2-year-old group. For the toddlers born to HBsAg-positive mothers, the proportion of delayed BCG vaccinations was significantly higher than that of the general pediatric population for both groups (*p* < 0.05, Table 3).

**Figure 1:**
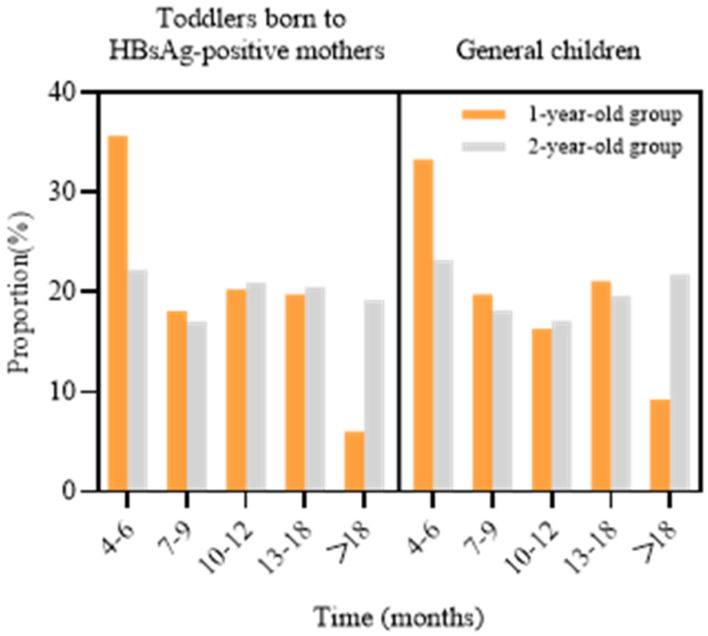
The distribution of delay time of BCG vaccination among toddlers born to HBsAg-positive mothers in 2021–2022.

In the cohort of toddlers born to HBsAg-positive mothers, 416 experienced BCG vaccination delays in 2021–2022, including 182 from the 1-year-old group and 234 from the 2-year-old group. The delay duration for the 1-year-old group was primarily 4–6 months (35.7%), while for the 2-year-old group, the delays were mostly 4–6 months (22.2%), 10–12 months (20.9%), and 13–18 months (20.5%). The distribution of delay duration was consistent in both toddlers born to HBsAg-positive mothers and the general pediatric population, with the majority of delays occurring within 4–6 months (Figure 1). In the general pediatric population, the duration of delayed BCG vaccination in the 1-year-old group was primarily 4–6 months (33.4%), while in the 2-year-old group, it was predominantly 23.2%.

### 3.4. Vaccination Status of Non-NIP Vaccines

The proportion of non-NIP vaccines among toddlers born to HBsAg-positive mothers ranged from 1.6% to 32.9% in 2021–2022. The proportion of children vaccinated with the first dose of DTaP-IPV/Hib was 27.0%; for PCV13, it was 36.4%; and for EV71, it was 35.1% (Table 4).

### 3.5. Influencing Factors Associated with the Timely Vaccination

In this study, a total of 3082 children received timely vaccination with HepB_1–3_, BCG, PV_1–3_, DTP_1–4_, MCV_1–2_, JEV-L_1_, MPV-A_1–2_, and HepA_1_, accounting for 72.5% of the 4250 individuals. Univariate analysis revealed that region level (χ^2^ = 4.620, *p* = 0.032), maternal HPV vaccination status (χ^2^ = 9.688, *p* = 0.002), and birth weight (χ^2^ = 5.227, *p* = 0.022) were statistically significant factors (*p* < 0.05) (Table 5).

**Figure 2:**
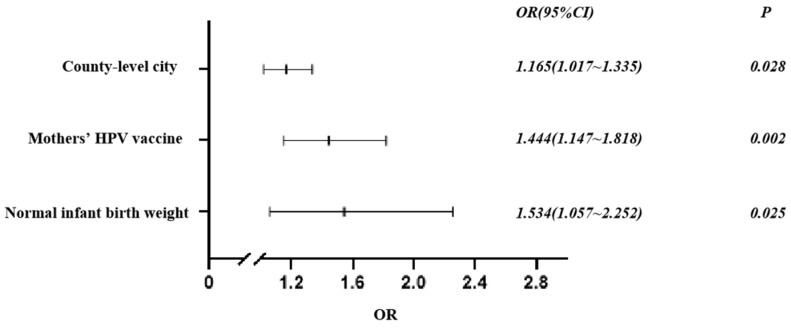
Factors associated with timely vaccination among toddlers born to HBsAg-positive mothers in 2021–2022.

Significant variables were subsequently selected for multivariate analysis as independent variables. The dependent variable was “timely vaccination,” which was categorized as “yes” or “no”. A binary logistic regression analysis was utilized for this analysis. The results indicated that living in a county-level city (OR = 1.165, 95% CI = 1.017–1.335), having mothers who received timely vaccinations (OR = 1.444, 95% CI = 1.147–1.818), and having a normal birth weight (OR = 1.534, 95% CI = 1.057–2.252) were factors that promoted timely vaccination (Figure 2).

## 4. Discussion

The World Health Organization has set a goal to eliminate viral hepatitis as a public health threat by 2030, with the aim of reducing new chronic hepatitis B infections by 90% [12]. The most effective method of preventing hepatitis B infection is vaccination with HepB, primarily targeting newborns and then young children. Early administration of HepB to infants born to HBsAg-positive mothers can effectively prevent mother-to-child transmission and reduce the risk of hepatitis B transmission [13]. In this study, all children received the first dose of HepB on time, and the completion rate for the full course was 98.8%, a figure that is in agreement with a similar study conducted in Zhejiang Province [14]. The protective efficacy of the hepatitis B vaccination was found to be high, even among exposed infants in Cameroon [15]. As HepB coverage has increased, the prevalence of hepatitis B surface antigen positivity in children under the age of 15 has decreased to 0.8% [16]. Other studies have suggested that hepatitis B vaccination coverage and awareness need to be improved amongst medical students and immigrants [17,18]. Therefore, timely HepB vaccination can reduce the burden of HBV and contribute to achieving the elimination goals set for 2030.

In this study, the coverage of NIP vaccines was over 90%, and the coverage was generally higher in the 2-year-old group than in the 1-year-old group. High age group coverage will be higher, and the trends are consistent with the results of a study in Australia [19]. Additionally, since the inclusion of the varicella vaccine in Suzhou City’s immunization program in 2018, the VarV coverage rate has been 95.0%, which was similar to that in Southern China. Since varicella vaccine licensure in 1995, approximately 90% of children in the United States have received two doses of the varicella vaccine, and the incidence of varicella has decreased dramatically [20].

The proportion of children born in Suzhou in 2021–2022 with delayed BCG vaccination was 5.2%. However, the delayed proportion of BCG-vaccinated children born to HBsAg-positive mothers was 11.3%, which was also higher than the children in the other groups [21]. Children with special health care needs (CSHCN) in temporarily unstable physiological conditions, such as premature birth, congenital diseases, and immunodeficiency, have gradually become a high-risk group for vaccine-preventable diseases. CSHCN have a low rate of universal vaccination and a high rate of delayed vaccination. A study of 657 children with congenital heart defects in Germany showed that only 34% of the children reached the full vaccination status, and the rate of HepB was lower than 80% [22]. Similarly, in another study in the United States, only 60% completed the full vaccination among 260 subjects [23]. Therefore, the special group of children born to HBsAg-positive mothers should be regarded. Infants were required to be promptly vaccinated with BCG and HepB_1_ after birth. HBsAg-positive mothers may pay more attention to HepB and delay BCG. In addition, delayed vaccination may increase the risk of early childhood illness, while BCG vaccination could make infants produce antibodies as early as possible. BCG vaccination prevents tuberculosis in adolescents and adults and is credited with having ended the tuberculosis epidemic [24]. In the study, the rate of BCG after catch-up vaccination was 98%, and the delay time focus was 4 to 6 months. In addition to the contraindications, this may be related to the children’s own conditions and the inaccurate judgment of outpatient medical staff. A study in Weija Gbawe Municipality showed that the coronavirus pandemic disrupted the expansion of their immunization program, and the BCG rate fell to 69.1% from 67.7% [25].

Non-NIP vaccinations followed the principle of voluntary, self-funded immunization, based on parent choice. The intention to receive non-NIP vaccines varied across regions, which may have been limited by public vaccine acceptance, income, and the impact of vaccine hesitancy. Our results showed that the proportion of children who received a first dose DTaP-IPV/Hib, PCV13, and EV71 was 3.3–36.4%, and the proportion of DTaP -IPV/Hib was 27.0%, higher than other research [26]. Compared with the other group of children, the DTaP-IPV/Hib vaccination rate of children born to HBsAg-positive mothers was higher. This may be related to the HBsAg-positive mothers being more attentive to the vaccine or having a rich knowledge reserve of vaccines. The DTaP-IPV/Hib vaccine was licensed in the USA in 2008, and a safety study found no risks associated with the combination DTaP-IPV/Hib vaccines [27].

Residing in a county-level city can facilitate the timely vaccination of infants, potentially due to population mobility and the service capabilities of vaccination clinics. Studies have shown that the timely vaccination rate of transient populations can be affected [28]. The interconnectedness and frequent population movement in all districts of Suzhou City may lead to delays in child vaccination. This study demonstrated that the timely vaccination rate was high among children born to mothers who received the HPV vaccine. The HPV vaccine is widely sought-after and often has a long waiting list. Mothers’ sustained attention to the HPV vaccine may reflect their emphasis on personal healthcare and their overall trust in vaccines. Additionally, a healthy birth weight can influence timely vaccination. Generally, newborns with a birth weight between 2500 g and 4000 g are considered to the normal birth weight, while those with a birth weight of less than 2500 g are considered to the low birth weight. Studies have shown that low birth weight is associated with vaccine delay, with lower-weight preterm neonates having a lower timely vaccination rate [29,30]. Parents’ knowledge of vaccines and accessibility to medical services are important factors affecting timely vaccination. Parents’ lack of vaccine-related knowledge will lead to delayed vaccination of children [31,32], such as concerns about vaccine efficacy, misconceptions about the immunization process, and adverse events following immunization. The higher the awareness of vaccine-preventable diseases among HBsAg-positive mothers, the more timely the vaccination of their children, which may be related to the higher awareness of parents. In addition, accessibility to medical services can also contribute to vaccine hesitancy, such as long distances between vaccination sites, complicated appointment procedures, and shortages of health care workers [33]. The presence of a large floating population makes it difficult to make appointments in vaccination clinics, which may also reduce parents’ willingness to bring their children for vaccination on time.

This study has some limitations, including a small sample size, which may limit its representativeness. Additionally, this study focused on children under the age of two, a demographic with high mobility, which may lead to errors in the acquisition of vaccination information.

## 5. Conclusions

In conclusion, while the vaccination status of toddlers born to HBsAg-positive mothers in Suzhou city remains stable, attention needs to be paid to instances of delayed vaccination. It is crucial to enhance targeted promotion of vaccine knowledge to reassure parents and increase vaccine acceptance. Additionally, the capabilities and diagnostic and treatment levels of vaccination outpatient services should be improved. Particularly for children in special circumstances, the issuance of relevant guidelines should help reduce missed vaccinations due to vaccine hesitancy.

## Figures and Tables

**Table 1:** Information of toddlers born to HBsAg-positive mothers in 2021–2022.

**Variables**	**Total**	**Proportion (%)**
Sex		
Male	2276	53.6
Female	1974	46.4
Age groups		
1-year-old group (2022.1.1–2022.12.31)	2048	48.2
2-year-old group (2021.1.1–2021.12.31)	2202	51.8
Region		
County-level	1940	45.6
District-level	2310	54.4
Grade of delivery hospital		
Secondary hospital	868	20.4
Tertiary hospital	3382	79.6
Birth weight		
Normal birth weight (2500–4000 g)	4130	97.2
Low birth weight (<2500 g)	120	2.8
Apgar score (1 min)		
≥8	4202	98.9
<8	48	1.1
Only child or not		
Yes	4182	98.4
No	68	1.6
Maternal age at delivery (years)		
≤25	92	2.2
26–30	726	17.1
31–35	1901	44.7
36–40	1252	29.5
>40	279	6.6

**Table 2:** The information of NIP vaccines of toddlers born to HBsAg-positive mothers in 2021–2022.

Vaccines_dose_	Proportion (%)			Proportion of Delayed Vaccination (%)		
	1-Year-Old Group	2-Year-Old Group	Total	1-Year-Old Group	2-Year-Old Group	Total
HepB_1_	100 (2048/2048)	100 (2202/2202)	100 (4250/4250)	0 (0/2048)	0 (0/2202)	0 (0/4250)
HepB_2_	100 (2048/2048)	99.7 (2196/2202)	99.9 (4244/4250)	0 (0/2048)	0.4 (8/2202)	0.2 (8/4250)
HepB_3_	99.8 (2043/2048)	98.0 (2157/2202)	98.8 (4200/4250)	1.0 (21/2048)	3.0 (67/2202)	2.1 (88/4250)
BCG	98.6 (2013/2041)	98.4 (2164/2199)	98.5 (4177/4240)	10.3 (210/2041)	12.2 (269/2199)	11.3 (479/4240)
PV_1_	100 (2047/2047)	98.8 (2175/2201)	99.4 (4222/4248)	0 (0/2047)	1.4 (31/2201)	0.7 (31/4248)
PV_2_	99.8 (2042/2047)	98.5 (2169/2201)	99.1 (4211/4248)	0.5 (11/2047)	2.3 (50/2201)	1.4 (61/4248)
PV_3_	99.0 (2026/2047)	97.6 (2148/2201)	98.3 (4174/4248)	2.6 (54/2047)	5.6 (123/2201)	4.2 (177/4248)
DTP_1_	99.9 (2044/2047)	98.6 (2172/2202)	99.2 (4216/4249)	0.2 (4/2047)	1.8 (39/2202)	1.0 (43/4249)
DTP_2_	99.7 (2040/2047)	98.1 (2161/2202)	98.9 (4201/4249)	0.7 (14/2047)	3.1 (68/2202)	1.9 (82/4249)
DTP_3_	99.1 (2028/2047)	97.6 (2150/2202)	98.3 (4178/4249)	2.6 (54/2047)	5.9 (131/2202)	4.4 (185/4249)
DTP_4_	-	92.9 (2046/2202)	92.9 (2046/2202)	-	11.9 (262/2202)	11.9 (262/2202)
MCV_1_	98.5 (2015/2045)	97.0 (2135/2201)	97.7 (4150/4246)	3.7 (76/2045)	8.6 (189/2201)	6.2 (265/4246)
MCV_2_	-	93.8 (2065/2201)	93.8 (2065/2201)	-	8.4 (184/2201)	8.4 (184/2201)
JEV-L_1_	98.0 (2004/2045)	96.7 (2129/2201)	97.3 (4133/4246)	5.2 (106/2045)	9.9 (217/2201)	7.6 (323/4246)
MPV-A_1_	99.3 (2031/2046)	98.5 (2169/2202)	98.9 (4200/4248)	0.6 (13/2046)	2.6 (58/2202)	1.7 (71/4248)
MPV-A_2_	96.3 (1971/2046)	96.1 (2116/2202)	96.2 (4087/4248)	3.9 (79/2046)	6.4 (142/2202)	5.2 (221/4248)
VarV	94.8 (1938/2045)	95.3 (2097/2201)	95.0 (4035/4246)	-	-	-
HepA	-	94.1 (2071/2202)	94.1 (2071/2202)	-	7.9 (173/2202)	7.9 (173/2202)

**Table 3:** The information of delayed of BCG vaccination among toddlers born to HBsAg-positive mothers in 2021–2022.

Population Group	1-Year-Old Group					2-Year-Old Group				
	Total	Number of People with Delayed Vaccination	Proportion of Delayed Vaccination (%)	χ^2^	*p*	Total	Number of People with Delayed Vaccination	Proportion of Delayed Vaccination (%)	χ^2^	*p*
Toddlers born to HBsAg-positive mothers	2041	210	10.3	101.655	<0.001	2199	269	12.2	167.096	<0.001
General children	91,475	4775	5.2			99,888	5692	5.7		

**Table 4:** The information of non-NIP vaccines among toddlers born to HBsAg-positive mothers in 2021–2022.

Vaccines	Proportion of First Dose (%)			Proportion of Full Vaccination (%)		
	1-Year-Old Group	2-Year-Old Group	Total	1-Year-Old Group	2-Year-Old Group	Total
DTaP-Hib	58 (2.8%)	82 (3.7%)	140 (3.3%)	12 (0.6%)	56 (2.5%)	68 (1.6%)
DTaP-IPV/Hib	624 (30.5%)	523 (23.8%)	1147 (27.0%)	278 (13.6%)	431 (19.6%)	709 (16.7%)
PCV13	797 (38.9%)	752 (34.2%)	1549 (36.4%)	595 (29.1%)	543 (24.7%)	1138 (26.8%)
EV71	706 (34.5%)	787 (35.7%)	1493 (35.1%)	645 (31.5%)	752 (34.2%)	1397 (32.9%)

**Table 5:** Factors associated with timely vaccination among toddlers born to HBsAg-positive mothers in 2021–2022.

Variables	Number of People with Timely Vaccination	Number of People with Delayed Vaccination	χ^2^	*p*
Sex				
Male	1632	644	1.625	0.202
Female	1450	524		
Region				
County-level	1438	502	4.620	0.032
District-level	1644	666		
Birth weight				
Normal birth weight (2500–4000 g)	3006	1124	5.227	0.022
Low birth weight (<2500 g)	76	44		
Apgar score (1 min)				
≥8	3049	1153	0.346	0.557
<8	33	15		
Only child or not				
Yes	3030	1152	0.542	0.462
No	52	16		
Maternal HPV vaccination status				
Yes	373	102	9.688	0.002
No	2709	1066		

## Data Availability

The data are not publicly available due to the sensitive nature of the information. The data will be made available from the corresponding author upon request.
